# Bone turnover in passive smoking female rat: relationships to change in bone mineral density

**DOI:** 10.1186/1471-2474-12-131

**Published:** 2011-06-11

**Authors:** Shu-guang Gao, Kang-hua Li, Mai Xu, Wei Jiang, Hong Shen, Wei Luo, Wen-shuo Xu, Jian Tian, Guang-hua Lei

**Affiliations:** 1 Department of Orthopaedics, Xiangya Hospital, Central South University, No.87 Xiangya Road, Changsha, Hunan, 410008, China; 2 Medical Research Center, Xiangya Hospital, Central South University, No.87 Xiangya Road, Changsha, Hunan, 410008, China; 3 Orthopaedics Institute of Central South University, No.87 Xiangya Road, Changsha, Hunan, 410008, China

## Abstract

**Background:**

Many studies have identified smoking as a risk factor for osteoporosis, but it is unclear whether passive smoking has an effect on bone mineral density and bone turnover and if such an effect could cause osteoporosis.The purpose of the study was to investigate the effect of passive smoking on bone mineral density (BMD) and bone turnover and the relationship between BMD and bone turnover in female rat.

**Methods:**

Forty-eight female Wistar rats were randomized into six groups: 2-month, 3-month,4-month smoke-exposed rats and their controls. A rat model of passive cigarette smoking was prepared by breeding female rats in a cigarette-smoking box for 2, 3 or 4 months. Serums were analyzed for levels of osteocalcin, bone-specific alkaline phosphatase (b-ALP) and Tartrate-resistant acid phosphatase 5b (TRACP 5b). BMD was assessed at lumbar vertebrae and femur by dual energy X-ray absorptiometry in passive smoking rats and in control rats.

**Results:**

BMD of lumbar spine and femur was lower in 4-month smoke-exposed female rats than that in controls. However, there was no significant difference in serum osteocalcin levels between smoke-exposed rats and controls. Significantly lower b-ALP and higher TRACP 5b were found in the 3-month or 4-month smoke-exposed rats compared to controls. Subsequent analysis showed that b-ALP positively correlated with BMD of the lumbar vertebrae(r = 0.764, P = 0.027) and femur(r = 0.899, P = 0.002) in 4-month smoke-exposed female rats. Furthermore, TRACP 5b levels negatively correlated with BMD of lumbar vertebrae (r = -0.871, P = 0.005) and femur (r = -0.715, P = 0.046) in 4-month smoke-exposed female rats.

**Conclusion:**

Our data suggest that smoke exposure can inhibit bone formation and increase bone resorption. The hazardous effects of passive smoking on bone status are associated with increased bone turnover in female rat.

## Background

Osteoporosis is a chronic, progressive disease of the skeleton characterized by bone fragility due to a reduction in bone mass and possibly alteration in bone architecture which leads to a propensity to fracture with minimum trauma[1]. Many studies found that smoking was a risk factor for osteoporosis[2-10]. Meta-analyses of the effects of smoking on bone status have demonstrated decreased bone mass in current smokers compared to non-smokers, although data for men was limited[11,12]. Ward et al. [12] reported that the decrease in bone mass of smokers was greater in men than in women. Additionally, smoking had more adverse effects on bone mass for individuals aged 60 years or more [12]. A review by Wong et al. [6] indicated that effect of smoking on bone mass appeared to be dose-dependent based on a meta-analysis [12]. Smoking was also associated with lower areal BMD (bone mineral density) and reduced cortical thickness in young men [13]. Smoking cessation, relative to continued smoking, increased BMD at the femoral trochanter and total hip in postmenopausal women[8].

Cigarette smoking is a worldwide public health problem. Cigarette smoke is composed of a large variety of substances, of which nitrogen, oxygen, and carbon dioxide account for 85%. Nicotine, which is one of the addictive components of tobacco, is a highly toxic alkaloid and has been the focus of several studies evaluating the relationship between specific cigarette components and bone. However, the effect of nicotine on bone remains controversial, with some studies finding adverse effects[14,15] and others showing no effects[16,17]. Compared to nicotine treatment alone, cigarette smoke exposure has been found to be more detrimental to bone[18], which suggests that cigarette smoke constituents (e.g., toxic heavy metals, polychlorinated biphenyls, dioxin, polycyclic aromatic hydrocarbons) other than nicotine might be responsible for the negative impact of smoking on bone. The mechanisms by which cigarette exerted its negative effect on bone are not fully understood.

There has been recent interest in the use of bone-turnover markers to evaluate osteoporosis. Bone turnover markers can be categorized as bone formation markers, measured in the serum, or bone resorption markers, measured in the serum or urine. Compston [19] showed that older smokers had high levels of bone resorption while early postmenopausal women had low levels of bone formation, though the mechanisms had not been clearly established. At present, the most sensitive markers for bone formation are serum osteocalcin, Bone-specific alkaline phosphatase (B-ALP) and procollagen type I N-terminal propeptide (PINP). Bone resorption can be assessed by several biochemical markers, N-terminal and C-terminal crosslinking telopeptides of type-I collagen (NTX-I and CTX-I), deoxypyridinoline (DPD) and TRACP 5b. Serum TRACP 5b reflects the number and activity of osteoclasts on bone surface. Serum TRACP 5b levels are elevated in patients with bone diseases and decreased in subjects on antiresorptive treatment, suggesting that serum TRACP 5b is a specific and sensitive marker of bone resorption[20-22].

It is unclear whether passive smoking has an effect on BMD and bone turnover, and if such an effect could cause osteoporosis. We hypothesized that passive smoking may have a negative effect on BMD in female rat by increasing bone turnover. To provide experimental proofs for this hypothesis and approach the possible mechanism of the association between smoking and osteoporosis, we examine the impact of smoking on bone turnover and BMD of the femur and lumbar vertebrae in a rat model of passive cigarette smoking. In this study, bone turnover was assessed by determining serum osteocalcin and b-ALP for bone formation and TRACP 5b levels for bone resorption.

## Methods

### Animals

Forty-eight 6-month-old female Wistar rats about 272 g were purchased from SLRC Laboratory Animal (Shanghai, China). The experiment was performed with the approval of the Animal Research Committee of the Xiangya Hospital, and the animals were cared for in the Experimental Animal Center of Xiangya Hospital.The rats were acclimated to conditions for one week before the experiment and randomly assigned to six groups of 8 rats each, three control groups and three smoke-exposed groups. The smoke-exposed groups were bred in an environment with smoking for 2, 3 or 4 months.These rats were housed at 25°C with a 12:12-hour light-dark cycle and had free access to standard laboratory food and tap water.

### Cigarette Smoking

The rats of passive smoking model were exposed to Sidestream cigarette smoke(CS) in an ashtray placed 10 cm below the rat cage in an exposure box (30 cm × 5 cm × 15 cm) made of polypropylene, which was placed in a laboratory draft chamber [23]. Using this equipment, the time, amount, and interval of smoke to be sent into the chamber was defined, and 10 cigarettes could be set at a time. The blood concentration of nicotine was measured to reflect the intensity of the smoking habit.The contents of the reference research cigarettes conform to the US Federal Trade Commission standards. Cigarette smoke was sent for 10 minutes and then the box was ventilated with room air for 10 minutes. This procedure was repeated 10 times at 1-hour intervals such that the amount of cigarette smoking per day in the rats was equal to in heavy smokers who smoke equal to 30 cigarettes per day [24].

### Blood nicotine concentrations and baseline bone density characteristics

The rats were sacrificed by intraperitoneal administration of sodium pentobarbital after 2, 3 or 4 months, and the lumbar spine (L4-L6) and both femurs were removed. Blood was collected from the abdominal vena cava into heparinized tubes and centrifuged at 1500 r.p.m. and 4°C for 5 min. Separated plasma was stored at -70°C for subsequent determination of blood nicotine concentrations. Nicotine concentrations in plasma were measured by gas chromatography with nitrogen-phosphorus detection. Sensitivity of the assay was 1 ng/ml nicotine. BMD of femurs and lumbar vertebrae (L4-L6) were measured by dual-energy X-ray absorptiometry (DXA; Hologic QDR-4500A) equipped with appropriate software for bone assessment in small animals[25]. The coefficients of variations (CV) for repeated measurements on the same bone was <1.0% for BMD. The results of the BMD in femur are expressed as the mean value of both femurs. Similarly to the femur, the results of the BMD in lumbar spine are expressed as the mean value of L4-L6.

### Biomarkers of bone turnover

Venous blood samples were drawn into serum separator tubes from all subjects. The serum was taken after a centrifugation under room temperature and stored at -70°C until it was assayed. Serum osteocalcin concentration was measured by an enzyme-linked immunosorbent assay (Rat Mid™Osteocalcin ELISA kit, IDS Inc., Fountain Hills, AZ, USA) according to the manufacturer's instructions. The intra- and inter-assay variations of this measurement in our laboratory were 6.8% and 8.9%, respectively. Serum b-ALP was measured by immunoassay using the Access Ostase Assay (Beckman Access, Beckman Coulter Inc., Fullerton, CA, USA). The intra-assay and interassay coefficients of variation were 4.1% and 5.2%, respectively. Serum TRACP 5b level was measured by a solid phase immunofixed enzyme activity assay (Rat TRACP™ Assay, IDS Inc., Fountain Hills, AZ, USA) according to the manufacturer's instructions to assess bone resorption. The intra- and inter-assay variations of this measurement in our laboratory were 4.9% and 7.3%, respectively.

### Statistical analysis

Results of all measurements were presented as mean ± S.D. for eight rats in each group. A one-way analysis of variance (ANOVA) was first performed to determine whether there were statistically significant (p < 0.05) differences among the experimental groups. Further, the Duncan's multiple range post-hoc test was used for comparisons between individual groups and to determine which means differed statistically significantly (p < 0.05). Pearson'correlation was applied to determine the correlation between markers of bone turnover and BMD of the femur and lumbar vertebrae. Correlations were considered statistically significant at p < 0.05. Data analysis was performed with SPSS version 13.0 (Chicago, IL, USA).

## Results

### Body weight

The rats were weighed weekly throughout the whole experiment. The initial weights and final weights are shown in Table 1. At the beginning and end of the protocol, there was no difference in weight between controls and smoke-exposed female rats (p > 0.05). All animals increased their weight during the experiment. The body weight gain of rats receiving passive smoking during the 2, 3, 4-month experimental period was similar to that noted in the control groups. The smoke exposure had no effect on the body weight of rats. Though individual food intake was not measured, total food supply in smoke-exposed groups was similar to that in the control groups. All animals in each group survived throughout the experiment and they showed no obvious clinical signs of morbidity.

**Table 1:** Initial and final weights in the controls and smoke-exposed rats.

*Group*	*initial weights(g)*	*final weights(g)*
2-month smoke-exposed rats	270 ± 23	286 ± 31
controls (2-month)	271 ± 28	291 ± 26
3-month smoke-exposed rats	280 ± 16	306 ± 27
controls (3-month)	271 ± 25	318 ± 29
4-month smoke-exposed rats	283 ± 17	344 ± 28
controls (4-month)	272 ± 26	362 ± 22

Values are means (for 8 rats) with their standard deviation.

### Blood nicotine concentrations

**Figure 1:**
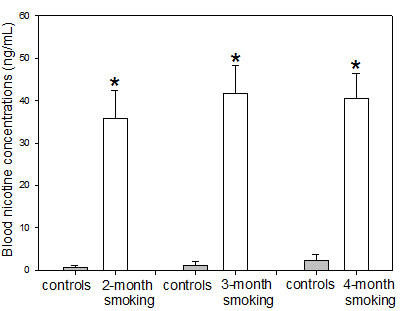
**Blood concentrations of nicotine**. Significantly higher blood nicotine concentrations were noted in the smoke-exposed rats compared to controls. Statistics: means ± SD (8 rats per group), *p < 0.05 in comparison to controls.

The blood nicotine concentrations in the 2-month, 3-month and 4-month smoke-exposed rats were 35.8 ± 6.7 ng/mL, 41.7 ± 6.5 ng/mL and 40.6 ± 5.9 ng/mL, respectively. The concentrations in their controls were 0.7 ± 0.5, 1.2 ± 0.9, 2.4 ± 1.3 ng/mL. The blood nicotine concentrations in the smoke-exposed rats were significantly higher than those in controls (p < 0.01) (Figure 1).

### Bone density characteristics

**Figure 2:**
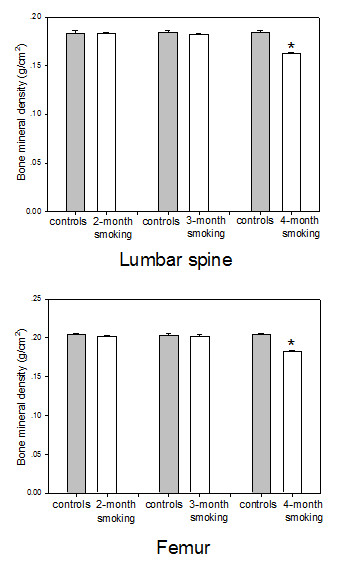
**Bone mineral density of the lumbar vertebrae and femur in the controls, 2-month, 3-month and 4-month smoke-exposed rats**. Bone mineral density of the lumbar vertebrae and femur in the 4-month smoke-exposed rats was significantly lower than that in controls. Mean ± SD (n = 8 in each group). *p < 0.05 compared to controls.

BMD of the lumbar vertebrae and femur are demonstrated in Figure 2. There was no significant difference between the lumbar spine BMD at 2-month or 3-month, but the lumbar spine BMD in the 4-month smoke-exposed rats was significantly lower than that in controls (p < 0.01) (Figure 2). Similarly to the lumbar spine, these data on the femur BMD did not differ significantly at 2-month or 3-month, but the BMD of the femur was significantly lower in the smoke-exposed rats at 4 months (p < 0.01) (Figure 2). Smoke exposure resulted in a decrease in the lumbar spine BMD by 11.8% and the femur BMD by 10.5% after 4 months, respectively, compared to control.

### Bone turnover

**Figure 3:**
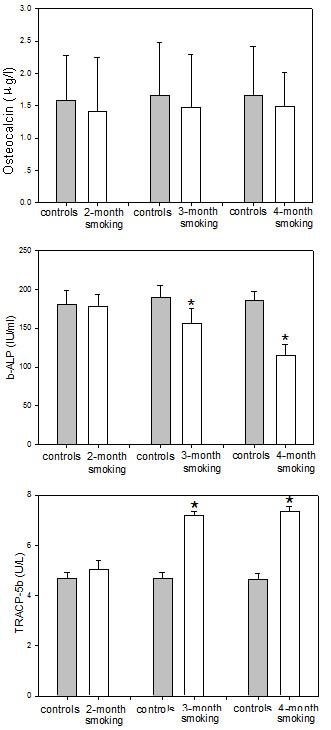
**Levels of osteocalcin, b-ALP and TRACP 5b**. There was no significant difference in serum osteocalcin levels between controls and smoke-exposed rats. Significantly lower b-ALP and higher TRACP 5b levels were found in the 3-month or 4-month smoke-exposed female rats compared to controls. Statistics: means ± SD (8 rats per group), *p < 0.05 in comparison to controls.

Figure 3 shows the results of the bone turnover measurements in the controls and smoke-exposed female rats. There was no significant difference in serum osteocalcin levels between smoke-exposed rats and controls(p > 0.05) (Figure 3). There was also no significant difference in the serum b-ALP activity and the TRACP 5b levels between 2-month smoke-exposed groups and controls(p > 0.05). However, significantly lower b-ALP activity and higher TRACP 5b levels were noted in the 3-month or 4-month smoke-exposed rats compared to controls (p < 0.01) (Figure 3). Smoke exposure resulted in a decrease in the serum b-ALP activity by 17.5% and 37.8% after 3 and 4 months, respectively, compared to control. Furthermore, passive smoking resulted in a increase in the TRACP 5b by 53.9% and 59.4% after 3 and 4 months, respectively, compared to control.

### Correlations between BMD and biomarkers of bone turnover

**Figure 4:**
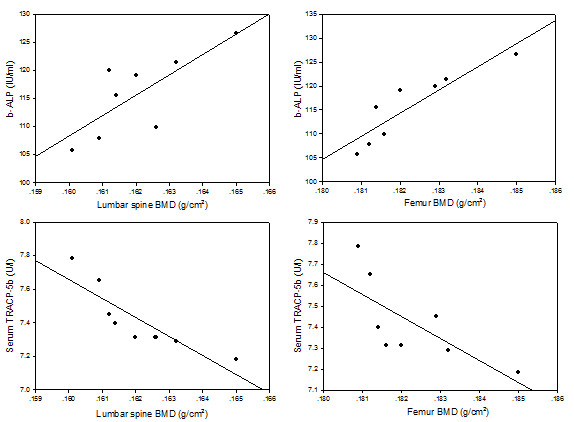
**The correlation between markers of bone turnover and BMD**. The b-ALP positively correlated with bone mineral density of the lumbar vertebrae (r = 0.764, P = 0.027) and femur (r = 0.899, P = 0.002) in 4-month smoke-exposed female rats. TRACP 5b level negatively correlated with bone mineral density of the lumbar vertebrae (r=-0.871, P = 0.005) and femur (r=-0.715, P = 0.046) in 4-month smoke-exposed female rats.

Interestingly, b-ALP positively correlated with BMD of the lumbar vertebrae (r = 0.764, P = 0.027, Figure 4) and femur (r = 0.899, P = 0.002, Figure 4) in 4-month smoke-exposed female rats. Furthermore, TRACP 5b level negatively correlated with BMD of the lumbar vertebrae (r = -0.871, P = 0.005, Figure 4) and femur (r = -0.715, P = 0.046, Figure 4) in 4-month smoke-exposed female rats.

## Discussion

Our finding revealed a significant effect of smoking characteristic on BMD of lumbar spine and femur in rat, consistent with the results from previous studies[26,27]. We demonstrated that the BMD of lumbar spine and femur was lower in 4-month smoke exposed rats than in controls, but not in the 2-month and 3-month smoke exposed rats, which may be due to differences in time of passive smoking. César-Neto et al.[26] showed that 5-month cigarette smoke inhalation promoted a reduced bone density. Hapidin et al. [28] demonstrated that 4 months of nicotine treatment was detrimental to bone by causing an increase in the bone resorbing cytokines and cotinine levels and nicotine also exerted negative effects on the dynamic trabecular histomorphometric parameters. Tamaki et al.[29] reported the impact of smoking on bone status was mainly associated with the number of smoking years in elderly men. Furthermore, Sneve et al.[30] showed that smoking reduced BMD at the hip, distal and ultradistal forearm in males, and the effect appeared to be mainly time- but not dose-dependent.

However, previous investigations also have shown that the timing of BMD decrease may be associated with the dose level of smoke exposure. Epping-Jordan et al. [24] reported the mean blood nicotine concentration for smokers who smoked 30 cigarettes daily was 40-42 ng/ml. Cornuz et al.[31] indicated that the risk of hip fracture increased linearly with greater cigarette consumption. It seems that in the case of high exposure, the effect of smoking develops very fastly and the exposure duration needed for developing of a measurable effect of smoking depends on its intensity. In this study, the blood nicotine concentrations in the 4-month smoke-exposed rats were 40.6 ng/ml, indicating that the average blood nicotine concentration was similar to the average for heavy smokers. The blood nicotine concentration did not differ among 2, 3 and 4 months, but there may be a harmful effect of longer smoke exposure for 4 months. Thus, we considered that the timing of BMD decrease may be associated with the dose level and the duration of smoke exposure. We could have shown a threshold dose if we examined an intermediate time point. It will be of interest to perform this analysis in a future study.

The skeleton in rats and humans responded similarly to mechanical influences, hormones, and other agents[32]. Therefore, it should be taken into account that the prevalence of the BMD values noted in the rat model of smoke exposure might be over-estimated compared to its prevalence in conditions of human exposure. Fukuda et al. [33] indicated that the rat may reach maturity in bone age at 17-21 weeks. They concluded that the rat at this age may be regarded as corresponding to a human adult of 18-24 years of age. Therefore, the 3-month-old rat is equivalent to a 13-year-old human, the 6-month-old rat corresponds to a 26-year-old human. Hapidin et al.[34] suggested nicotine administration for 2 months approximately corresponded to 8 years of smoking in humans, while 4 months of nicotine administration was equivalent to 17 years of smoking in humans. Therefore, our study implicated that continuous exposure to cigarette smoke for 17 years may be harmful to BMD of the human skeleton. That is to say, for 26-year-old adult who smoked 30 cigarettes daily, it will be very possible for her to have a low BMD at the age about 43.

Previous studies of the effects of smoking on osteocalcin have also produced conflicting results, possibly because the number of subjects studied has been relatively small[35-37]. Nielsen et al. [35] and Tamaki et al. [29] reported that there was no effect of smoking on osteocalcin in humans. Ortego-Centeno et al. [36] and Supervia et al. [37] found that there were no different values of serum osteocalcin between smokers and non-smokers in studies with young men. Gürlek et al. [38] indicated that osteocalcin levels in smoker patients was lower than the controls. Reduced serum osteocalcin levels have also been reported in early postmenopausal women who smoked[39]. The data on effect of smoke exposure on osteocalcin in rats are completely lacking. In this study, smoking did not seem to decrease osteocalcin level in rats, consistent with the results of previous report[29,35-37]. It remains unclear why the osteocalcin level was not decreased during the whole smoke exposure, in spite of decreased activity of b-ALP in serum.

The document on the effect of smoke exposure on b-ALP or TRACP 5b are very rare. Broulik et al.[40]reported nicotine-induced bone loss was associated with high bone turnover in the male rats as expressed by increased b-ALP and TRACP. Supervia et al [37] reported there were no effect of smoking on serum TRACP. We demonstrated that smoke exposure decreased activity of b-ALP and increased the level of TRACP 5b, suggesting that smoking inhibited bone formation and increased bone resorption. Our findings about TRACP 5b, which had a low diurnal variability and varied significantly with time of smoke exposure in female rats, would indicate that passive smoking can increase osteoclast activities. Increased rate of bone turnover with the rate of bone resorption exceeding that of bone formation can lead to bone loss. In addition, b-ALP positively correlated with BMD of the lumbar vertebrae and femur in 4-month smoke-exposed female rats. Furthermore, it is also of interest that changes in TRACP 5b levels negatively correlated with BMD of the femur and lumbar vertebrae in 4-month smoke-exposed female rats. These results suggest that the hazardous effect of smoking on bone status is associated with increased bone turnover in female rat. Our finding revealed the peak of BMD at the femur and lumbar vertebrae was achieved later than that of b-ALP or TRACP 5b. Thus, the smoking effect on BMD might be seen even when value for the signal-to-noise ratio of BMD is lower than those of b-ALP or TRACP 5b.

While many studies had focused on the hazardous effects of smoking on the skeleton, there was little direct evidence regarding which substance in cigarette exerted this effect. The major components of the smoke may affect the skeleton included carbon monoxide [41], nicotine[14] and other toxic components of cigarettes[42-45] (e.g., Cadmium, Lead, Chromium, Benzo(a)pyrene, dimethylbenz(a)anthracene). Loder et al.[41] reported that the incidence of congenital spinal deformations was directly related to carbon monoxide dose. In a previous study, nicotine given in the drinking water for 3 months was found to lower BMC in male mice [14]. Syversen et al.[46]showed that female Sprague Dawley rats were exposed to nicotine vapour for 2 years and there was no difference in BMD between control rats and nicotine-exposed rats. Berley et al.[47] and Yamano et al.[48] indicated that systemic nicotine may have a significant adverse impact on bone wound healing and inhibit the bone matrix-related gene expressions required for wound healing. Brzóska et al. [42,43] indicated that low exposure to cadmium (Cd) may affect the mineralization and biomechanical properties of growing bone, thus increasing the risk of fractures. Sankaramanivel et al.[44] revealed that a significant increase in the concentration of Chromium can decrease bone formation rate. Benzo(a)pyrene (BaP) and 7,12-dimethylbenz(a)anthracene (DMBA) are polycyclic aromatic hydrocarbons (PAHs) found in the tar fraction of cigarette smoke. According to Lee et al. [45], BaP and DMBA can cause a loss of bone mass and bone strength, possibly through an increase in bone turnover. Their results revealed that BaP and DMBA, two PAHs found in the tar fraction of cigarette smoke, may be in part responsible for the adverse effect of cigarette smoking on the bone[45].

Previous studies found that passive smoking caused some 8 percent of 18,000 female deaths and 1 percent of 49,000 male deaths from lung cancer, as well as 9 percent of 34,000 female deaths and 4 percent of 42,000 male deaths from ischemic heart disease[49], which suggests that women are the main victims of passive smoking. We confirmed that passive smoking increased the risk of bone damage in female rat. Thus, our findings allow for the conclusion that passive smoking may increase the risk of bone damage in women.

Female may be more susceptible than male to the disease caused by tobacco. It is well established that the bone susceptibility to damage is to a high extent determined by a gender, and if regarding the bone effect in men and women the future study should involve both male and female rats. Furthermore, comparison of impact of passive smoking on the bones in female rat with in male rat is also necessary.

Though the study was aimed to investigate the impact of passive smoking, examining the effects of active smoking on bone was also valuable. Difficulties existed in drawing a direct comparison to smoking in humans, since humans inhale tobacco directly from cigarettes. Thus, the model used in the present study reflected the effects of passive cigarette smoking, but not those of active smoking. Humans may be active smokers or passive smokers, and we cannot comment on the effects of active smoking using our model.

In addition, cigarette smoke was composed of many different substance. In this study, the rats were exposed to whole cigarette smoke and not just to a specific substance of cigarette alone. We did not demonstrated which kinds of substance in cigarette exactly exerted the hazardous effect. Another limitation was a relatively small sample size in this study. The analyses based on a greater sample size were necessary for a more definitive conclusion.

## Conclusion

In summary, the present study suggests that passive smoking increases bone turnover and then decreases bone mineral density and the change in BMD is associated with increasing bone turnover in female rat.

## Competing interests

The authors declare that they have no competing interests.

## Authors' contributions

SGG, KHL and GHLwere involved in the conceptual discussion and design of the study. SGG carried out the biochemical analyses. MX, WJ and WL performed the statistical analysis. SGG and GHL drafted the manuscript. WSX and JT were involved in the data collection. KHL and GHL obtained the funding for National Natural Science Foundation of China. All authors readed and approved the final manuscript.

## Pre-publication history

The pre-publication history for this paper can be accessed here:

http://www.biomedcentral.com/1471-2474/12/131/prepub
